# Accuracy of Contrast-Enhanced Mammography (CEM) Compared With Breast MRI in Detecting Residual Disease After Neoadjuvant Therapy: A Systematic Review

**DOI:** 10.7759/cureus.115429

**Published:** 2026-08-30

**Authors:** Yousef Alalawi, Sultan Abdulrahman S Alamrani, Mashael S Alharbi

**Affiliations:** 1 Department of Surgery, King Salman Armed Forces Hospital, Tabuk, SAU; 2 Department of Radiology, King Fahad Specialist Hospital, Tabuk, SAU

**Keywords:** breast cancer, breast mri, cem, cesm, contrast-enhanced mammography, contrast-enhanced spectral mammography

## Abstract

Accurate assessment of residual tumor burden following neoadjuvant therapy (NAT) is critical for predicting patient outcomes and guiding surgical decision-making in breast cancer management. While breast MRI remains the reference standard for posttreatment evaluation, contrast-enhanced mammography (CEM) has emerged as a promising alternative because MRI is often limited by its high cost, limited accessibility, prolonged examination time, and contraindications in certain patient populations. This study aims to evaluate the diagnostic precision of breast MRI and CEM in identifying residual disease in patients with breast cancer following neoadjuvant treatment. Using PubMed/MEDLINE, Embase, Cochrane CENTRAL, and Web of Science, a systematic review was carried out between January 2021 and January 2026 in accordance with the PRISMA standards. Included studies were prospective or retrospective head-to-head comparative studies reporting sufficient information to create 2 × 2 contingency tables for precise diagnosis, with histopathology as the reference standard. Six studies met the eligibility criteria, comprising 349 participants. The Quality Assessment of Diagnostic Accuracy Studies-2 (QUADAS-2) tool was used to assess quality. MRI sensitivity varied from 44.4% to 100%, whereas CEM sensitivity ranged from 77.8% to 100%. MRI specificity varied from 47% to 94.4%, whereas CEM specificity ranged from 50% to 83.3%. CEM demonstrated a tendency toward higher sensitivity but lower specificity compared with MRI. The remaining tumor size was consistently overestimated by both modalities by approximately 1-3 mm. False-negative rates were notably low for CEM (0-22.2%) but reached 55.6% for MRI in one small study. Identification of residual ductal carcinoma in situ remained challenging for both techniques, with improved detection when incorporating mammographic calcification assessment. The QUADAS-2 assessment indicated a low risk of bias for most studies. When identifying residual disease following neoadjuvant treatment, CEM and MRI exhibit similar diagnostic accuracy, with MRI having better specificity and CEM having higher sensitivity. When MRI is unavailable or contraindicated, CEM is a dependable substitute because of its practical benefits, which include reduced cost, shorter examination times, greater availability, and the lack of gadolinium-related contraindications. Tumor subtype, patient features, and institutional resources should all be taken into consideration when selecting a modality.

## Introduction and background

With an estimated 2.3 million new cases and 685,000 deaths in 2020, breast cancer remains the most frequently diagnosed malignancy among women worldwide, accounting for 11.7% of all cancer diagnoses [1]. Neoadjuvant treatment, administered prior to definitive surgical resection, has been the standard of care for locally advanced breast cancer over the past two decades and is increasingly utilized in high-risk, early-stage disease [2]. The primary objectives of neoadjuvant therapy (NAT) include downstaging the primary tumor to facilitate breast-conserving surgery, evaluating in vivo tumor response to systemic therapies, and providing critical prognostic information based on the extent of pathological response. Pathological complete response (pCR), most commonly defined as the absence of residual invasive carcinoma in both the breast and axillary lymph nodes (ypT0 ypN0), is strongly associated with improved event-free and overall survival, particularly in aggressive molecular subtypes [3]. Advances in targeted therapies have yielded pCR rates of approximately 60-70% for human epidermal growth factor receptor 2 (HER2)-positive disease, 40-50% for triple-negative breast cancer, but only 10-20% for luminal-type tumors [4].

Currently, breast MRI with IV gadolinium-based contrast material is the gold standard imaging modality for assessing residual disease following NAT [5]. Its superior soft-tissue contrast, multiplanar capabilities, and functional information derived from dynamic contrast-enhanced sequences enable detailed characterization of tumor vascularity, morphology, and residual enhancement patterns. A major meta-analysis of 44 studies, including 2,152 patients, reported pooled sensitivity and specificity of 79% (95% CI, 71-85%) and 80% (95% CI, 75-85%), respectively, with an area under the summary receiver operating characteristic curve of 0.86 [6]. However, several significant limitations restrict its widespread clinical utility, including high costs, prolonged examination times (30-45 minutes), limited accessibility in low-resource and community settings, and contraindications such as severe renal impairment (risk of nephrogenic systemic fibrosis), claustrophobia, certain implantable devices, and prior allergic reactions to gadolinium-based agents [7]. Moreover, even in patients with normal renal function, emerging evidence of gadolinium deposition in human tissues, including the dentate nucleus and bone, has raised long-term safety concerns [8].

In this context, contrast-enhanced mammography (CEM), also known as contrast-enhanced spectral mammography (CESM), has emerged as a promising alternative to breast MRI. CEM is a dual-energy mammographic technique that employs IV iodinated contrast material (typically 1.5 mL/kg at 3 mL/s) to acquire low- and high-energy images, generating both conventional mammographic and subtracted contrast-enhanced views that depict tumor angiogenesis [9]. Compared with MRI, CEM offers several practical advantages: shorter acquisition time (approximately 10 minutes), lower cost (roughly one-third to one-half that of MRI), broader availability (requiring only a software and hardware upgrade to existing digital mammography systems), and avoidance of gadolinium-related safety concerns [10]. Preliminary meta-analyses have suggested that CEM may achieve comparable diagnostic performance to MRI in the post-NAT setting, with similar specificity and no statistically significant difference in overall accuracy.

Nevertheless, a dedicated, head-to-head systematic comparison of breast MRI and CEM specifically for detecting residual disease after NAT, with sensitivity and specificity as primary outcomes, has not been updated in recent years. While existing meta-analyses have evaluated each modality separately or in mixed populations, none have focused exclusively on paired, direct comparative studies in the post-neoadjuvant setting. Therefore, the aim of this systematic review is to comprehensively evaluate and compare the diagnostic accuracy (sensitivity and specificity) of breast MRI and CEM for identifying residual disease in breast cancer patients following NAT, thereby clarifying their relative roles in contemporary clinical practice.

## Review

Methods

Search Strategy

Following the PRISMA guidelines [11], a thorough systematic literature search was carried out to identify studies comparing the diagnostic accuracy of breast MRI and CEM for identifying residual disease in patients with breast cancer following neoadjuvant treatment. The electronic databases PubMed/MEDLINE, Embase, Cochrane Central Register of Controlled Trials (CENTRAL), and Web of Science were searched. Only studies published within the previous five years (January 2021 to January 2026) were included in the search. Medical Subject Headings (MeSH) and free-text phrases were used in the search strategy, including “contrast-enhanced mammography”, “CEM”, “contrast-enhanced spectral mammography”, “CESM”, “breast MRI”, “magnetic resonance imaging”, “neoadjuvant therapy”, “neoadjuvant chemotherapy”, “residual disease”, “pathological complete response”, “pCR”, “sensitivity”, and “specificity”. Search phrases were combined using Boolean operators (AND, OR).

Eligibility Criteria

Studies that satisfied the following predetermined eligibility requirements based on the PICOS framework were included in this systematic review: (1) Population: adult female patients (age ≥18 years) who underwent definitive breast surgery after completing NAT (chemotherapy, endocrine therapy, or targeted therapy) for histopathologically proven primary breast cancer; (2) Index test: CEM, including CESM, performed following completion of neoadjuvant treatment; (3) Comparator: breast MRI with gadolinium-based contrast enhancement performed in the same patients following completion of neoadjuvant treatment; (4) Outcomes: sensitivity and specificity for identifying residual disease (invasive carcinoma or ductal carcinoma in situ, DCIS) compared with the histopathological reference standard were the primary outcomes; false-negative rate (FNR) and false-positive rate (FPR), positive predictive value (PPV) and negative predictive value (NPV), and the relationship between imaging and pathological tumor size were the secondary outcomes; (5) Study design: head-to-head comparison studies, either prospective or retrospective, that provided sufficient information to create 2 × 2 contingency tables for diagnostic accuracy. The following were excluded: (1) studies that assessed CEM or MRI alone without a direct head-to-head comparison; (2) case reports, case series with fewer than 10 patients, editorials, conference abstracts without full text, and reviews; (3) studies that did not use histopathology as the reference standard; (4) studies in which imaging was performed prior to completion of NAT was not completed; and (5) duplicate publications or overlapping cohorts.

Study Selection

Rayyan Intelligent Systematic Review (Rayyan Systems Inc., Cambridge, MA, USA), a collaborative online tool for managing systematic reviews, was used for the study selection process [12]. All retrieved records were first imported into Rayyan, where duplicate entries were automatically identified and manually confirmed for deletion. The titles and abstracts were screened against the eligibility criteria by two independent reviewers. A full-text review was conducted for any record that at least one reviewer considered potentially relevant. For final inclusion, the full-text publications were evaluated independently by the same two reviewers. At any point, disagreements between reviewers were resolved by discussion or, if consensus could not be achieved, by consulting a third senior reviewer.

Data Extraction

The full-text publications were evaluated independently for final inclusion by the same two reviewers. At any point, disagreements between reviewers were resolved by discussion or, if consensus could not be achieved, by consulting a third senior reviewer. Consensus or referral to a third reviewer was used to resolve any disagreements.

Two primary categories were created from the retrieved data. The first category included first author name, year of publication, place of origin, and study design (prospective or retrospective); sample size (number of lesions and participants); patient demographics (mean age, age range, and, if stated, menopausal status); tumor characteristics (histological type, hormone receptor status, HER2 status, and molecular subtype); NAT regimen; definition of pCR used; timing of post-NAT imaging relative to surgery; and reference standard details. The second category contained diagnostic accuracy outcomes: true-positive, false-positive, true-negative, and false-negative counts for both CEM and MRI; calculated sensitivity, specificity, PPV, NPV, accuracy, false-negative rate (FNR = 1 - sensitivity), and FPR (1 - specificity); correlation coefficients between pathological tumor size and imaging (e.g., Pearson’s r, Kendall’s tau, intraclass correlation coefficient, or concordance correlation coefficient); and any other reported parameters, including the area under the receiver operating characteristic curve (AUC). Data were designated as “not mentioned” (NM) when they were not included in the abstract or full text. Due to time restrictions, corresponding authors were not contacted regarding missing data.

Quality Assessment and Risk of Bias

Two reviewers independently evaluated the methodological quality and risk of bias of the included studies using the Quality Assessment of Diagnostic Accuracy Studies-2 (QUADAS-2) tool, which is specifically recommended for systematic reviews of diagnostic accuracy studies [13]. Four major domains are assessed by the QUADAS-2 tool: (1) patient selection; (2) index test (CEM); (3) reference standard (histopathology); and (4) flow and timing. The QUADAS-2 guidance’s signaling questions were used to classify each domain as having a “low risk,” “high risk,” or “unclear risk” of bias. Applicability concerns regarding the reference standard, index test, and patient selection were also assessed as “low,” “high,” or “unclear.” A study was categorized as having “low overall risk” if all domains were low, “high overall risk” if any domain was high, and “unclear overall risk” if at least one domain was unclear and no domain was high. Each study’s overall risk of bias was calculated by combining the domain ratings. Reviewers reached a consensus to resolve disagreements. A narrative summary and tabular presentation of the quality assessment results were provided. QUADAS-2 has demonstrated strong inter-rater reliability and content validity in previous systematic studies of CEM and MRI for breast cancer assessment.

Data Synthesis and Statistical Analysis

A meta-analysis was not carried out because of the expected clinical and methodological heterogeneity among the included studies (differences in sample size, pCR definitions, imaging techniques, and outcome reporting). Rather, a narrative synthesis of the diagnostic accuracy results was carried out. For each study, sensitivity, specificity, PPV, NPV, accuracy, FNR, and FPR were extracted or calculated for both CEM and MRI. These metrics were presented in tabular form alongside 95% confidence intervals when available. The direction and magnitude of differences between CEM and MRI were compared descriptively across studies. The results were tabulated and qualitatively analyzed for studies that reported correlation coefficients between imaging and pathological tumor size.

Results

Figure 1 shows the PRISMA flow diagram and outlines the systematic screening and selection process for studies included in the review. An initial database search yielded 931 records, of which 341 duplicates were removed, leaving 590 records for screening. After excluding 298 records at the title/abstract level, 292 reports were sought for retrieval, but 49 were unobtainable, leaving 243 full-text reports assessed for eligibility. These were excluded for wrong outcomes (n = 112), wrong population (n = 79), or being abstracts only (n = 46), ultimately resulting in six studies meeting the inclusion criteria for the final review.

**Figure 1:**
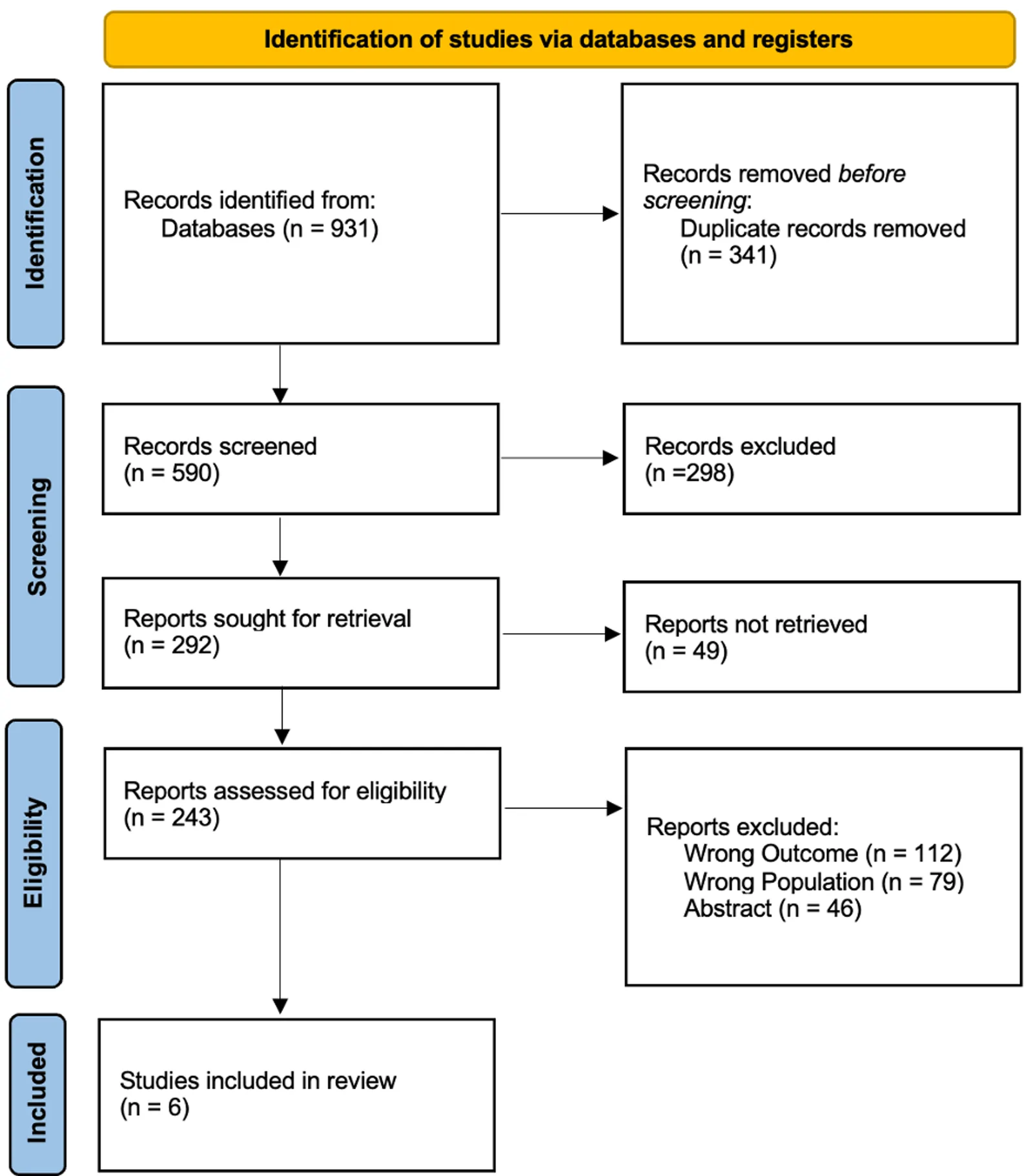
PRISMA flow diagram of study selection for systematic review.

Table 1 highlights the salient features of the six included studies that contrasted breast MRI with CEM for identifying residual disease following neoadjuvant treatment [14-19]. All studies were prospective in design, with sample sizes ranging from 14 patients (16 cancers) in Savaridas et al. [16] to 110 participants (115 cancers) in Hogan et al. [14]. Women with biopsy-proven breast cancer undergoing neoadjuvant chemotherapy or neoadjuvant treatment were included in the majority of studies, with mean ages reported only in Wang et al. [15] (47 ± 10 years) and Bernardi et al. [18] (46 ± 11 years). Geographic distribution included the USA [14], China [15], the UK [16], Italy [18], and Spain [19]; one study did not specify the country [17]. pCR definitions varied: Bernardi et al. [18] used the strictest definition (absence of both invasive cancer and DCIS), while Hogan et al. [14] defined residual disease as any invasive cancer or DCIS. All studies used histopathology as the reference standard, and post-NAT imaging timing ranged from within seven days [17] to an unspecified interval after NAT completion [14,15]. Notable differences included the multireader design in Wang et al. [15] and Savaridas et al.’s integration of digital breast tomosynthesis with CEM [16], which did not improve accuracy.

**Table 1:** Characteristics of included studies. CEM, contrast-enhanced mammography; CESM, contrast-enhanced spectral mammography; DBT, digital breast tomosynthesis; DCIS, ductal carcinoma in situ; LEI, low-energy images; pCR, pathological complete response; RI, recombined images

Study	Country/institution	Study design	Sample size (n)	Sample type	Patient age (mean ± SD/range)	Cancer type/subtype	NAT regimen	pCR definition	Imaging timing post-NAT	Reference standard	Notes
Hogan et al. (2023) [14]	USA (Memorial Sloan Kettering Cancer Center)	Prospective	110 participants, 115 breast cancers	Consecutive women undergoing NAT for breast cancer	NM (prospective enrollment July 2017 to July 2019)	Invasive cancer or DCIS	NM	Residual disease (invasive cancer or DCIS)	After completion of NAT	Histopathology	Two readers independently assessed MRI and CEM; CEM RI and LEI evaluated
Wang et al. (2025) [15]	China (Fudan University Shanghai Cancer Center)	Prospective, multireader	57	Patients undergoing CEM and MRI before and after NAT	47 ± 10 years	Breast cancer (subtype NM)	NM	pCR (no definition in abstract, but standard definition)	After NAT	Histopathology	Three readers with different experience levels independently assessed images
Savaridas et al. (2023) [16]	UK	Prospective	14 patients, 16 cancers	Women receiving neoadjuvant chemotherapy for breast cancer	NM	Breast cancer	Neoadjuvant chemotherapy	pCR (no further definition in abstract)	Post-neoadjuvant chemotherapy	Pathology specimens	Also evaluated the addition of DBT to CESM
Acar et al. (2026) [17]	NM	Prospective	74	Female patients with histopathologically confirmed breast cancer scheduled for neoadjuvant chemotherapy followed by surgery	NM	Breast cancer	Neoadjuvant chemotherapy	Absence of residual invasive carcinoma in surgical specimen	Within seven days post-neoadjuvant chemotherapy (surgery median 14 days after imaging)	Histopathology	CEM estimated residual tumor size within ±1 cm of histopathology in 84.7% vs MRI 76.4%
Bernardi et al. (2022) [18]	Italy	Prospective	51	Individuals who are candidates for NAT and have biopsy-proven breast cancer	46 ± 11 years	Breast cancer	NAT	Absence of both invasive cancer and DCIS	Pre-NAT, mid-NAT, post-NAT	Histopathology	Post-NAT CEM included six-minute delayed acquisition; one radiologist per modality
López et al. (2023) [19]	Spain	Observational, prospective	43	Patients with breast cancer treated with neoadjuvant chemotherapy	NM	Breast cancer	Neoadjuvant chemotherapy	NM	After neoadjuvant chemotherapy	Postoperative pathology	Presurgical study included both CEM and MRI

Table 2 presents the diagnostic accuracy outcomes for CEM vs MRI, with sensitivity and specificity as primary outcomes and FNR, FPR, predictive values, and lesion size correlation as secondary outcomes. Sensitivity values for CEM ranged from 77.8% [15] to 100% [16], while MRI sensitivity ranged from 44.4% [16] to 100% [18]. Bernardi et al. [18] reported significantly higher sensitivity for MRI (100%) compared with CEM (81%, p = 0.001), whereas Savaridas et al. [16] paradoxically found CEM sensitivity of 100% vs MRI sensitivity of only 44.4%, likely because of the small sample size (n = 16). Specificity showed an inverse pattern: MRI generally demonstrated higher specificity (85.7-94.4%) compared with CEM (50-83.3%), with the exception of Hogan et al. [14], in which CEM specificity (50%) slightly exceeded MRI specificity (47%). FNRs (1 - sensitivity) were notably low for CEM in Savaridas et al. [16] (0%) and Acar et al. [17] (8.7%), while MRI FNR reached 55.6% in Savaridas et al. [16] and 28.6% in Wang et al. [15]. FPR (1 - specificity) were generally higher for CEM, reaching 50% in Hogan et al. [14], compared with an MRI FPR as low as 5.6% in Wang et al. [15].

**Table 2:** Diagnostic accuracy outcomes of CEM vs. MRI for detecting residual disease. CEM, contrast-enhanced mammography; CESM, contrast-enhanced spectral mammography; DBT, digital breast tomosynthesis; FNR, false-negative rate; FPR, false-positive rate; ICC, intraclass correlation coefficient; LEI, low-energy images; NAT, neoadjuvant therapy; NM, not mentioned; NPV, negative predictive value; pCR, pathologic complete response; PPV, positive predictive value; RECIST, Response Evaluation Criteria in Solid Tumors; RI, recombined images

Study	Modality	Sensitivity (%)	Specificity (%)	Accuracy (%)	PPV (%)	NPV (%)	FNR (1 - sensitivity)	FPR (1 - specificity)	Lesion size correlation (with pathology)	Additional findings
Hogan et al. (2023) [14]	CEM (RI + LEI)	81	50	72	81	50	19%	50%	τ = 0.33	No significant differences between CEM and MRI (p ≥ 0.99); CEM detected residual disease in 55/115 (48%) on RI and 75/115 (65%) on LEI
	MRI (combined with CEM LEI)	88	47	77	81	60	12%	53%	τ = 0.36	MRI detected residual disease in 71/115 (62%)
Wang et al. (2025) [15]	CEM	77.8	71.4	75.4	NM	NM	22.20%	28.60%	ICC = 0.4467	CEM showed higher sensitivity and lower specificity vs MRI; pCR rate 36.8% (21/57); both overestimated residual extent (CEM: 1.7 mm, MRI: 2.7 mm)
	MRI	71.4	94.4	86	NM	NM	28.60%	5.60%	ICC = 0.449	MRI had higher accuracy and specificity; all P values > 0.05
Savaridas et al. (2023) [16]	CEM (CESM enhancement)	100	57.1	81.3	NM	NM	0%	42.90%	Concordance coeff = 0.70 (invasive size)	Greatest accuracy for pCR prediction with CESM enhancement; MRI sensitivity 44.4%, specificity 85.7%; DBT did not improve accuracy; CESM underestimated size, MRI overestimated (both p > 0.05)
	MRI	44.4	85.7	62.5	NM	NM	55.60%	14.30%	Concordance coeff = 0.66 (invasive size)	Small sample size (16 cancers)
Acar et al. (2026) [17]	CEM	91.3	NM (MRI slightly higher)	NM	NM	Higher than MRI	8.70%	NM	Agreement higher for CEM than MRI	CEM estimated size within ±1 cm of pathology in 84.7% vs MRI 76.4%; pCR rate 31.1%; MRI specificity slightly higher but not significant (p = 0.24)
	MRI	NM (lower than CEM, exact value not stated)	Slightly higher than CEM	NM	NM	Lower than CEM	NM	NM	Lower agreement vs pathology	CEM showed numerically higher sensitivity and NPV; full text needed for complete MRI data
Bernardi et al. (2022) [18]	CEM (post-NAT)	81	83	NM	NM	NM	19%	17%	Mean overestimation vs pathology: 0.8 mm	pCR rate 31.4% (16/51); MRI sensitivity significantly higher (p = 0.001); delayed CEM: sensitivity 81%, specificity 89%
	MRI (post-NAT)	100	86	NM	NM	NM	0%	14%	Mean overestimation vs pathology: 1.2 mm	CEM and MRI comparable for lesion size and RECIST categories (kappa 0.871)
López et al. (2023) [19]	CEM	83.9	83.3	NM	92.9	66.7	16.10%	16.70%	ICC = 0.9	CEM showed higher ICC than MRI (0.9 vs 0.7); CEM NPV 66.7% vs MRI NPV 57.9%; PPV similar (92.9% vs 95.8%)
	MRI	74.2	91.6	NM	95.8	57.9	25.80%	8.40%	ICC = 0.7	MRI had higher specificity and PPV

Regarding lesion size correlation, both modalities tended to overestimate residual tumor extent. Bernardi et al. [18] revealed a mean overestimation of 1.2 mm for MRI and 0.8 mm for CEM compared with pathology. Wang et al. [15] found that the agreement of CEM and MRI with pathology was low (intraclass correlation coefficients of 0.4467 and 0.449, respectively). Conversely, López et al. [19] reported higher correlation for CEM (ICC = 0.9) than MRI (ICC = 0.7), and Acar et al. [17] found that, in 84.7% of instances, CEM approximated residual tumor size within ±1 cm of histology, compared with 76.4% for MRI. Predictive values were reported in four studies: Hogan et al. [14] found identical PPVs for CEM and MRI (81% each), while López et al. [19] reported a CEM NPV of 66.7% vs an MRI NPV of 57.9%, suggesting that CEM may be better at ruling out residual disease. Acar et al. [17] similarly noted a higher NPV for CEM. Overall, while MRI shows superior specificity, CEM demonstrates comparable or higher sensitivity in several studies, supporting its potential suitability as a substitute for MRI when MRI is unavailable or contraindicated.

Table 3 presents the risk-of-bias assessment using the QUADAS-2 tool, which is widely used for systematic reviews of diagnostic accuracy studies. The included studies’ overall methodological quality ranged from moderate to excellent, with all studies using a prospective design and histopathology as the reference standard, which minimizes verification and reference standard bias. The domain-level risk-of-bias and applicability assessments are presented graphically in Figures 2, 3.

**Table 3:** Risk of bias assessment using the QUADAS-2 tool. QUADAS-2, Quality Assessment of Diagnostic Accuracy Studies-2

Study	Domain 1: Patient selection	Domain 2: Index test (CEM)	Domain 3: Reference standard (histopathology)	Domain 4: Flow and timing	Overall risk of bias	Applicability concerns
Hogan et al. (2023) [14]	Low	Low	Low	Low	Low	Low
Wang et al. (2025) [15]	Low	Low	Low	Low	Low	Low
Savaridas et al. (2023) [16]	Low	Low	Low	Low	Low	Low
Acar et al. (2026) [17]	Low	Low	Low	Low	Low	Low
Bernardi et al. (2022) [18]	Low	Low	Low	Low	Low	Low
López et al. (2023) [19]	Some concerns	Low	Low	Low	Some concerns	Some concerns

**Figure 2:**
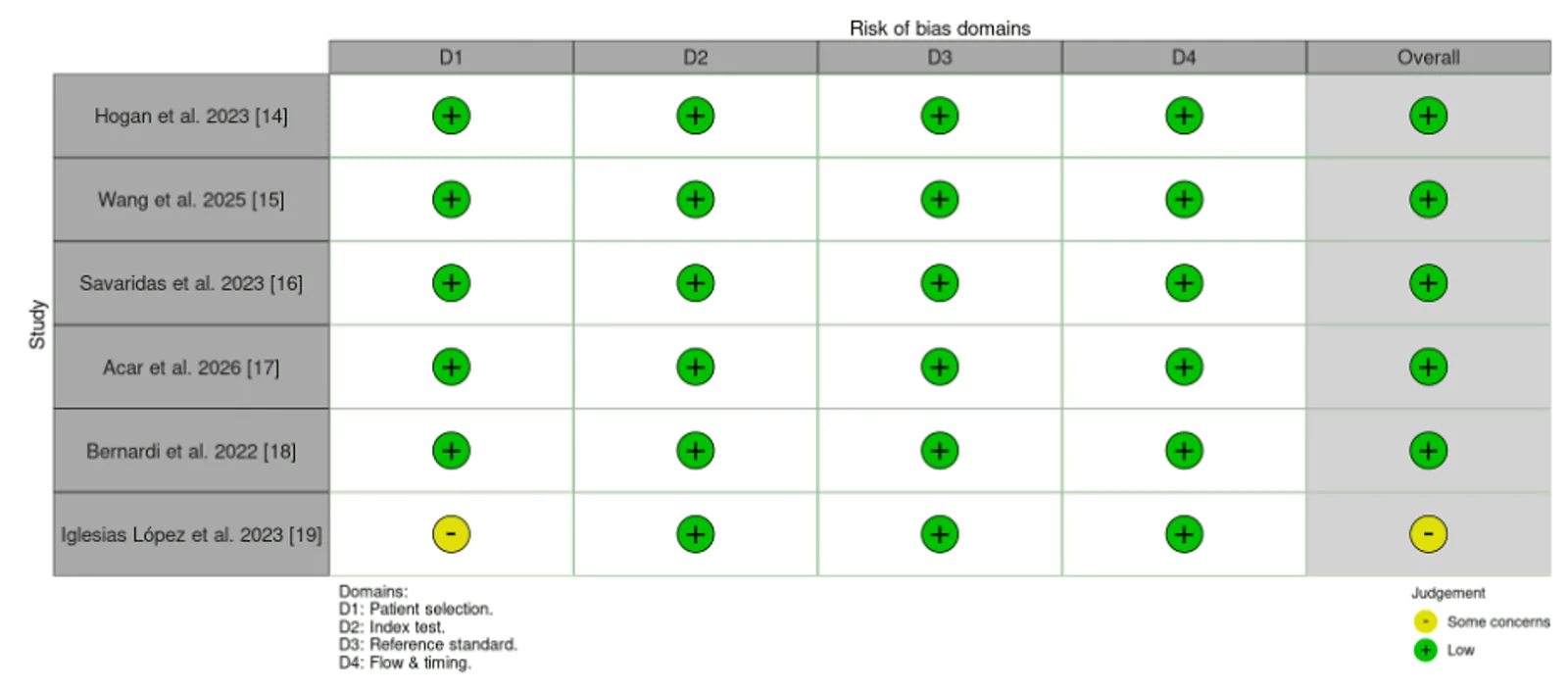
Risk of bias assessment using the QUADAS-2 tool. QUADAS-2, Quality Assessment of Diagnostic Accuracy Studies-2

**Figure 3:**
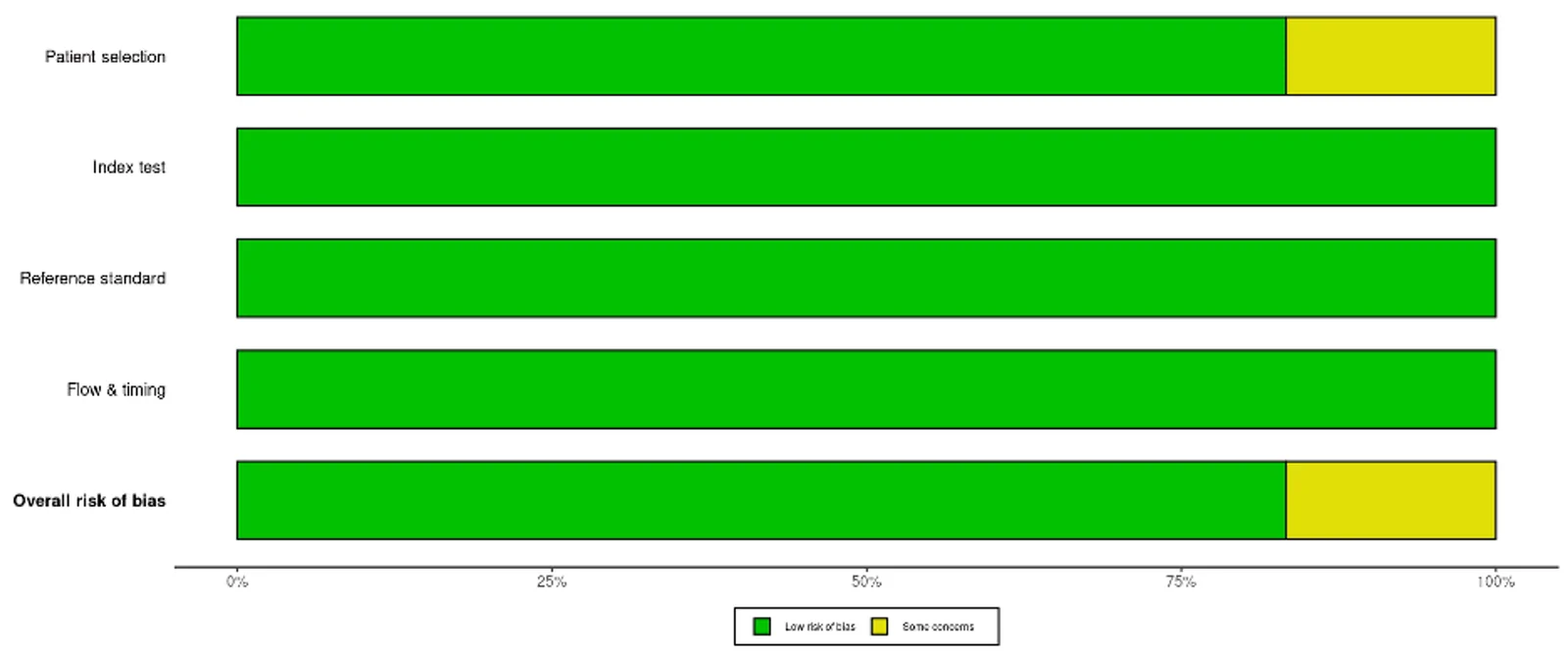
Risk of bias assessment using the QUADAS-2 tool. QUADAS-2, Quality Assessment of Diagnostic Accuracy Studies-2

Discussion

The diagnostic accuracy findings from our study demonstrate that CEM and MRI exhibit largely comparable performance, with CEM showing a tendency toward higher sensitivity (range 77.8-100% across studies), while MRI demonstrates superior specificity (range 85.7-94.4% across studies) [14-19]. These results are in line with the pooled results of a recent meta-analysis by Kaiyin et al., which included data from six head-to-head investigations involving 328 patients and found that the pooled sensitivity for CEM was 93% (95% CI, 84-97%) compared with 84% (95% CI, 62-95%) for contrast-enhanced MRI, and the pooled specificity was 68% (95% CI, 60-76%) for CEM vs 80% (95% CI, 71-87%) for CE-MRI, with both modalities having the same AUC of 0.85 [20]. Taken together, these aggregated data provide evidence that CEM may be noninferior to MRI for assessing post-NAT residual disease, with CEM potentially offering a sensitivity advantage at the expense of lower specificity [21].

The sensitivity and specificity trade-off observed between CEM and MRI in the present review warrants detailed discussion. In the Hogan et al. study, the largest included study with 115 breast cancers, MRI achieved higher sensitivity (88% vs 81% for CEM) but lower specificity (47% vs 50% for CEM), although these differences were not statistically significant after multiple-comparison adjustment (p ≥ 0.99) [14]. Wang et al. reported a more pronounced pattern: CEM sensitivity of 77.8% vs MRI sensitivity of 71.4%, and CEM specificity of 71.4% vs MRI specificity of 94.4% [15]. This inverse relationship between sensitivity and specificity reflects fundamental differences in how the two modalities characterize residual disease. With its better spatial resolution and soft-tissue contrast, MRI tends to detect more subtle enhancing lesions, leading to higher sensitivity but also more false-positive findings, particularly in the setting of post-NAT inflammatory changes, fibrosis, and granulation tissue that can mimic residual tumor. Conversely, CEM, which relies on iodine-based contrast agent distribution, may miss small-volume residual disease (lower sensitivity) but demonstrate fewer false-positive interpretations (higher specificity) in several studies [15,19]. The short investigation by Savaridas et al. (n = 16 cancers) found an exception to this tendency, with CESM enhancement showing 100% sensitivity and 57.1% specificity, while MRI showed only 44.4% sensitivity and 85.7% specificity, a striking reversal likely attributable to the very small sample size and possible selection bias rather than a true biological difference [16].

The impact of breast cancer tumor subtype on imaging performance is a crucial factor to take into account when evaluating these diagnostic accuracy statistics. The Harris et al. abstract reported on 397 patients (254 with both CEM and MRI) at the 2024 San Antonio Breast Cancer Symposium [8]. The results showed that overall sensitivity was 74% for CEM vs 81% for MRI, and specificity was 83% vs 71%, respectively, with CEM demonstrating significantly higher specificity (p = 0.02) [8]. Importantly, this large study stratified performance by tumor subtype, revealing that diagnostic accuracy varied according to biological characteristics; CEM was more effective in HER2-positive and triple-negative cancers, which are the subtypes most likely to achieve pCR following NAT [8]. This finding is consistent with the findings of Canteros et al., who found that patients with hormone receptor-negative tumors had greater CEM diagnostic accuracy [22]. The biological basis for this subtype-dependent performance likely relates to differences in tumor angiogenesis and contrast enhancement kinetics. Higher microvessel density and stronger contrast enhancement are usually seen in more aggressive subtypes (triple-negative and HER2-positive) [21], rendering them more conspicuous on both CEM and MRI, whereas luminal-type tumors often demonstrate more variable and sometimes weaker enhancement [23,24]. For clinical practice, this suggests that CEM may be particularly well suited for monitoring response in biologically aggressive breast cancers in which achieving pCR is both more likely and more clinically impactful.

A recurring challenge identified across multiple studies is the accurate assessment of residual DCIS. Bernardi et al. found that only seven (58%) of the 12 patients with residual DCIS had an enhancing lesion on post-NAT CEM, eight (67%) on MRI, and nine (75%) on delayed CEM acquisition [18]. This suggests that a significant percentage of residual DCIS does not exhibit contrast enhancement on either modality [18]. Only 57% of residual DCIS showed enhancement across 20 MRI studies, and the systematic review and meta-analysis by Ploumen et al., which included 31 studies, confirmed that calcifications on mammography can persist even after DCIS has completely responded [21]. This meta-analysis also showed that the definition of pCR has a significant impact on diagnostic performance: pooled sensitivity for MRI was higher (0.86 vs 0.82) when DCIS was considered pCR (ypT0/is), but pooled specificity was lower (0.61 vs 0.68) when residual DCIS was considered a non-pCR (ypT0) [21]. Hogan et al. similarly reported that CEM low-energy images (LEI), which include mammographic calcifications, detected residual disease in 75/115 (65%) lesions, whereas CEM recombined images, which depict enhancement only, detected only 55/115 (48%) lesions, highlighting the complementary value of evaluating both enhancement patterns and calcifications [14]. Savaridas et al. found that inclusion of residual microcalcifications improved concordance with whole tumor size (including DCIS), with correlation coefficients increasing from 0.69 (CESM enhancement alone) to 0.86 when microcalcifications were included [16]. These findings underscore a critical practical consideration: for optimal assessment of residual disease, particularly when DCIS may be present, imaging protocols should incorporate both contrast-enhanced sequences and evaluation of mammographic calcifications, regardless of whether CEM or MRI is employed.

Regarding residual tumor size estimation, the six included studies consistently showed that both CEM and MRI tend to overestimate pathological tumor size, with no statistically significant difference between modalities. Bernardi et al. found that, compared with pathology, the mean overestimation was 0.8 mm for CEM, 1.2 mm for MRI, and 1.9 mm for delayed CEM acquisition [18]. Wang et al. found that CEM overestimated residual extent by a mean of 1.7 mm and MRI by 2.7 mm, with both modalities exhibiting low pathological consistency (ICC of 0.4467 for CEM and 0.449 for MRI) [15]. Acar et al. found that, in 84.7% of instances, CEM assessed residual tumor size within ±1 cm of histology, compared with 76.4% for MRI, with greater agreement for CEM [17]. Ermans et al.’s systematic review and meta-analysis, which included 21 studies with 1,060 patients, found that both MRI and CEM showed a mean overestimation of tumor size by approximately 3 mm compared with pathology, with correlation coefficients of 0.78 for MRI and 0.79 for CEM, and strong direct agreement between the two modalities (R² = 0.81) [9]. This consistent tendency toward overestimation reflects the inability of any imaging modality to definitively distinguish between microscopic residual tumor, inflammatory changes, fibrosis, and treatment-related edema. From a surgical planning perspective, slight overestimation is generally preferable to underestimation, as it reduces the risk of positive margins; however, clinicians should be aware that both modalities systematically overestimate residual disease extent [9,18].

The practical implications of these findings are substantial, particularly given the clinical and economic context of breast cancer care. Breast MRI is associated with high costs, limited accessibility, longer examination times (typically 30-45 minutes), patient claustrophobia, gadolinium-based contrast agent contraindications (such as severe renal impairment or a history of allergic reactions), and difficulties in patients with implanted devices [4,5,25]. CEM offers several practical advantages: shorter examination time (approximately 10 minutes), lower cost, wider availability (requiring only a software and hardware upgrade to existing digital mammography systems), better patient tolerance, and absence of gadolinium-related safety concerns [4,5,25]. A review by Cozzi et al. reported that CEM exhibits sensitivity comparable to MRI for primary breast cancer detection (97% vs 97%), with 14% higher specificity (66% vs 52%) [26]. The diagnostic performance of CEM for post-NAT assessment, as demonstrated in this systematic review and supported by meta-analyses [20], supports its potential use as a practical substitute when MRI is unavailable, not recommended, or intolerable. However, several caveats warrant consideration: CEM involves exposure to ionizing radiation (approximately equivalent to a standard diagnostic mammogram), requires IV iodinated contrast with associated risks of allergic reaction and renal toxicity, and has fewer longitudinal outcome data compared with the well-established body of evidence supporting MRI [4,5]. Furthermore, the specificity advantage of MRI in several studies [15,16] and MRI’s superior soft-tissue characterization capabilities may make it the better option in situations where accurate evaluation of pectoralis muscle involvement, chest wall invasion, or axillary nodal disease is required.

The potential contribution of artificial intelligence and radiomics to improving the diagnostic accuracy of both modalities is an emerging topic of interest that was not specifically covered in the included research. Zhang et al.’s meta-analysis reported that radiomics based on dynamic contrast-enhanced MRI achieved a pooled sensitivity of 81% (95% CI, 73-87%) and specificity of 74% (95% CI, 54-88%) for predicting pCR, with an AUC of 0.83 (95% CI, 0.80-0.86) [10]. Preliminary studies suggest that CEM-based radiomics may offer similar predictive capabilities, potentially enabling early identification of nonresponders who could benefit from treatment modification [23,24]. To determine clear noninferiority margins between CEM and MRI for post-NAT response evaluation, future research should concentrate on standardizing acquisition techniques, creating validated radiomic signatures for CEM, and conducting extensive multicenter prospective studies.

Study Limitations

It is important to recognize the many limitations of this systematic study. First, the sample sizes of the six included studies varied significantly, ranging from 16 malignancies in Savaridas et al. [16] to 115 cancers in Hogan et al. [14]. Additionally, some studies defined pCR as the absence of invasive disease alone [17,18], whereas others included the absence of both DCIS and invasive malignancy [14,18]. The observed variability in sensitivity and specificity values may be attributed to this difference in definitions, which also affects the comparability of diagnostic accuracy estimates between studies. Second, radiologists were not fully blinded to clinical or pathological data in any of the included investigations, which might introduce expectation bias. Third, the absence of standardized thresholds for defining radiological complete response (e.g., residual enhancement size or qualitative enhancement assessment criteria) limits the generalizability of the findings and precludes formal meta-analysis of pooled diagnostic accuracy from the six studies. Fourth, all included studies were conducted in academic tertiary referral centers with high-volume breast imaging services, potentially limiting generalizability to community or low-resource settings where CEM may be most needed as an MRI alternative. Finally, the clinical importance of imaging-detected residual disease vs pCR could not be associated with patient outcomes because the included studies did not describe the follow-up duration for evaluating long-term outcomes (e.g., recurrence-free survival or overall survival).

## Conclusions

When identifying residual disease in patients with breast cancer following neoadjuvant treatment, CEM and breast MRI have similar diagnostic accuracy. Compared with MRI, CEM often has higher sensitivity but lower specificity. Both modalities systematically overestimate residual tumor size by approximately 1-3 mm, which may be clinically acceptable for surgical planning purposes. Detection of residual DCIS remains challenging for both techniques, with a substantial proportion of DCIS showing no contrast enhancement on either CEM or MRI; however, incorporation of mammographic calcification assessment (via CEM LEI or standard mammography) improves detection. CEM is a desirable alternative to MRI in settings with limited resources or for patients with MRI contraindications because of its practical benefits, which include lower cost, shorter examination time, greater availability, and avoidance of gadolinium-related contraindications. However, MRI maintains advantages in specificity and soft-tissue characterization. Future large-scale multicenter prospective trials with standardized pCR definitions, blinded interpretation protocols, and long-term outcome correlation are needed to establish definitive noninferiority margins. Until then, the choice between CEM and MRI for post-neoadjuvant response assessment should be individualized based on patient characteristics, tumor subtype, institutional resources, and specific clinical questions, with CEM representing a reliable alternative rather than a universal replacement for MRI.

## References

[1] Sung H, Ferlay J, Siegel RL, Laversanne M, Soerjomataram I, Jemal A, Bray F (2021). Global Cancer Statistics 2020: GLOBOCAN estimates of incidence and mortality worldwide for 36 cancers in 185 countries. CA Cancer J Clin.

[2] Korde LA, Somerfield MR, Carey LA (2021). Neoadjuvant chemotherapy, endocrine therapy, and targeted therapy for breast cancer: ASCO guideline. J Clin Oncol.

[3] Cortazar P, Zhang L, Untch M (2014). Pathological complete response and long-term clinical benefit in breast cancer: the CTNeoBC pooled analysis. Lancet.

[4] Spring LM, Fell G, Arfe A (2020). Pathologic complete response after neoadjuvant chemotherapy and impact on breast cancer recurrence and survival: a comprehensive meta-analysis. Clin Cancer Res.

[5] Mann RM, Cho N, Moy L (2019). Breast MRI: state of the art. Radiology.

[6] Houssami N, Macaskill P, von Minckwitz G, Marinovich ML, Mamounas E (2012). Meta-analysis of the association of breast cancer subtype and pathologic complete response to neoadjuvant chemotherapy. Eur J Cancer.

[7] Nieto Alvarez I, Madl J, Becker L, Amft O (2025). Patients' experience to MRI examinations—a systematic qualitative review with meta-synthesis. J Magn Reson Imaging.

[8] Harris W, Patel B, Kosiorek H (2025). Diagnostic accuracy assessing residual disease in breast cancer patients receiving neoadjuvant systemic therapies (NST) is comparable between contrast enhanced mammography(CEM)& breast magnetic resonance imaging(MRI)across various cancer subtypes. Cancer Res.

[9] Ermans SJ, Maas R, Hill TM, Usiskin SI, Alic L, Douek M (2026). Comparison of contrast-enhanced mammography and breast MRI in tumour extent assessment: A systematic review and meta-analysis. Eur J Surg Oncol.

[10] Zhang J, Li L, Zhe X, Tang M, Zhang X, Lei X, Zhang L (2022). The diagnostic performance of machine learning-based radiomics of DCE-MRI in predicting axillary lymph node metastasis in breast cancer: a meta-analysis. Front Oncol.

[11] Page MJ, McKenzie JE, Bossuyt PM (2021). The PRISMA 2020 statement: an updated guideline for reporting systematic reviews. BMJ.

[12] Ouzzani M, Hammady H, Fedorowicz Z, Elmagarmid A (2016). Rayyan—a web and mobile app for systematic reviews. Syst Rev.

[13] Lee J, Mulder F, Leeflang M, Wolff R, Whiting P, Bossuyt PM (2022). QUAPAS: an adaptation of the QUADAS-2 tool to assess prognostic accuracy studies. Ann Intern Med.

[14] Hogan MP, Horvat JV, Ross DS (2023). Contrast-enhanced mammography in the assessment of residual disease after neoadjuvant treatment. Breast Cancer Res Treat.

[15] Wang S, Li J, Chen R (2025). Head‑to‑head comparison of contrast‑enhanced mammography and MRI in assessing the tumor response to neoadjuvant therapy in breast cancer: a prospective, multireader study. Radiol Med.

[16] Savaridas SL, Vinnicombe SJ, Warwick V, Evans A (2023). Predicting the response to neoadjuvant chemotherapy. Can the addition of tomosynthesis improve the accuracy of contrast-enhanced spectral mammography? A comparison with breast MRI. Br J Radiol.

[17] Acar O, Açar ÇR, Orguc İS, Ekinci F, Sahbazlar M, Erdoğan AP (2026). Contrast-enhanced mammography vs. breast MRI for assessing neoadjuvant chemotherapy response: a prospective clinical comparison study. Diagnostics (Basel).

[18] Bernardi D, Vatteroni G, Acquaviva A (2022). Contrast-enhanced mammography versus MRI in the evaluation of neoadjuvant therapy response in patients with breast cancer: a prospective study. AJR Am J Roentgenol.

[19] López ÁI, Alejandro AB, López LA (2023). Diagnostic accuracy of contrast-enhanced mammography vs MRI in evaluating the response of breast cancer treated with neoadjuvant chemotherapy. Rev Senol Patol Mamaria.

[20] Kaiyin M, Lingling T, Leilei T, Wenjia L, Bin J (2023). Head-to-head comparison of contrast-enhanced mammography and contrast-enhanced MRI for assessing pathological complete response to neoadjuvant therapy in patients with breast cancer: a meta-analysis. Breast Cancer Res Treat.

[21] Ploumen RA, de Mooij CM, Gommers S, Keymeulen KB, Smidt ML, van Nijnatten TJ (2023). Imaging findings for response evaluation of ductal carcinoma in situ in breast cancer patients treated with neoadjuvant systemic therapy: a systematic review and meta-analysis. Eur Radiol.

[22] Canteros D, Walbaum B, Córdova-Delgado M (2022). Contrast-enhanced mammography predicts pathological response after neoadjuvant chemotherapy in locally advanced breast cancer. Ecancermedicalscience.

[23] Vidali S, Irmici G, Depretto C (2024). Performance of contrast-enhanced mammography (CEM) for monitoring neoadjuvant chemotherapy response among different breast cancer subtypes. Cancers (Basel).

[24] Sunen I, Isabel Garcia Barrado A, Cruz Ciria S, Garcia Maroto J, Gros Bañeres B, Garcia Mur C (2024). Is contrast-enhanced mammography (CEM) an alternative to MRI in assessing the response to primary systemic therapy of breast cancer?. Eur J Radiol.

[25] Patel BK, Gray RJ, Pockaj BA (2017). Potential cost savings of contrast-enhanced digital mammography. AJR Am J Roentgenol.

[26] Cozzi A, Schiaffino S, Sardanelli F (2019). The emerging role of contrast-enhanced mammography. Quant Imaging Med Surg.

